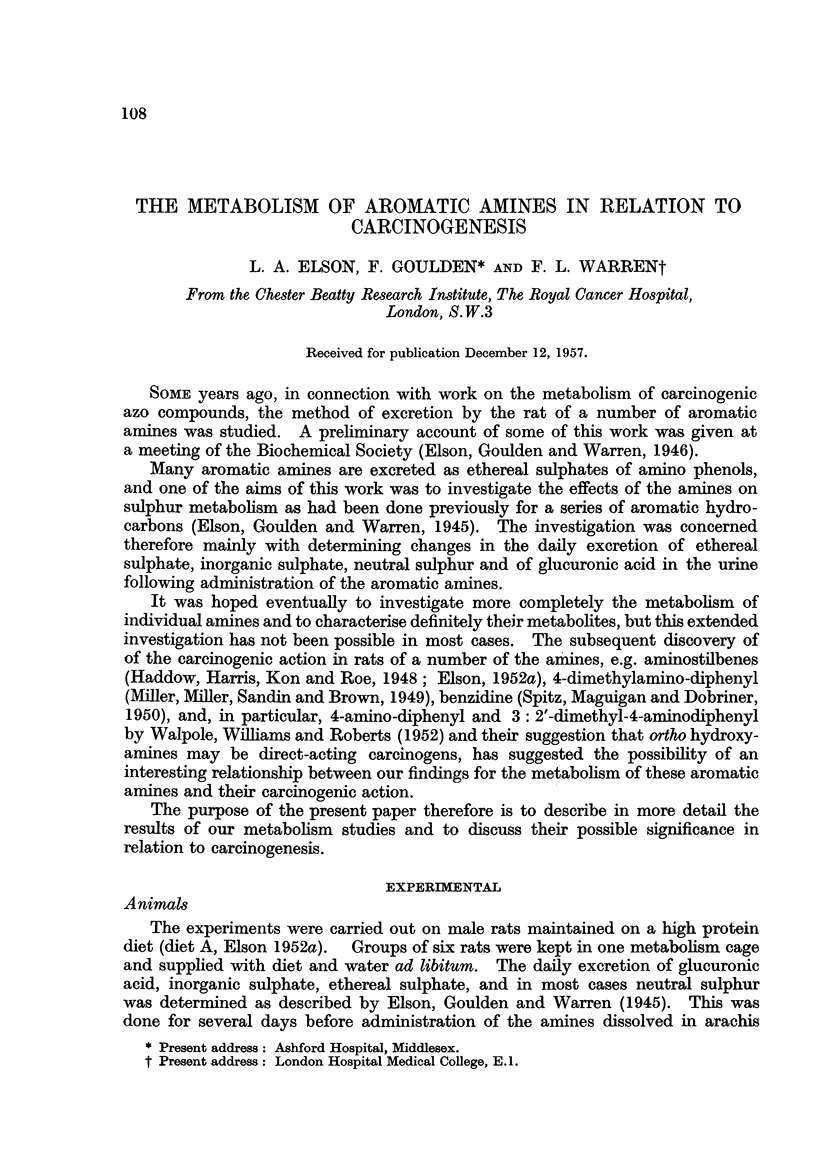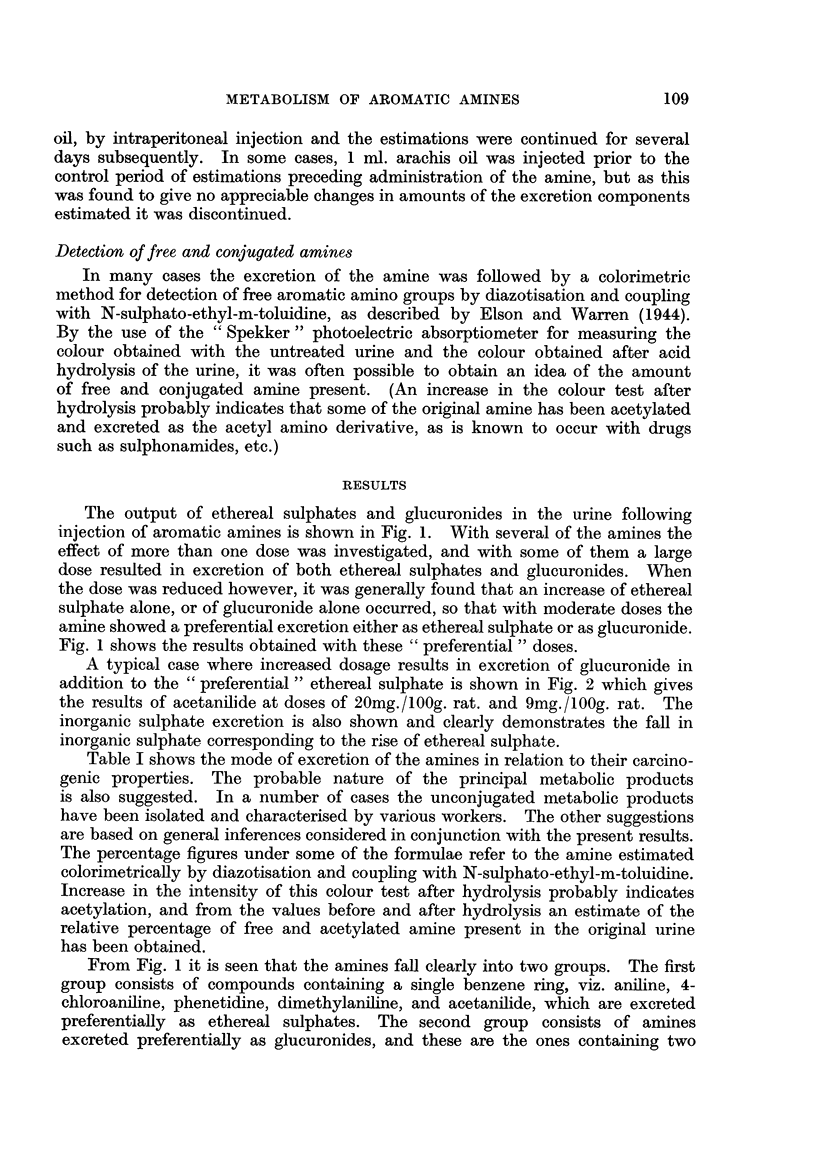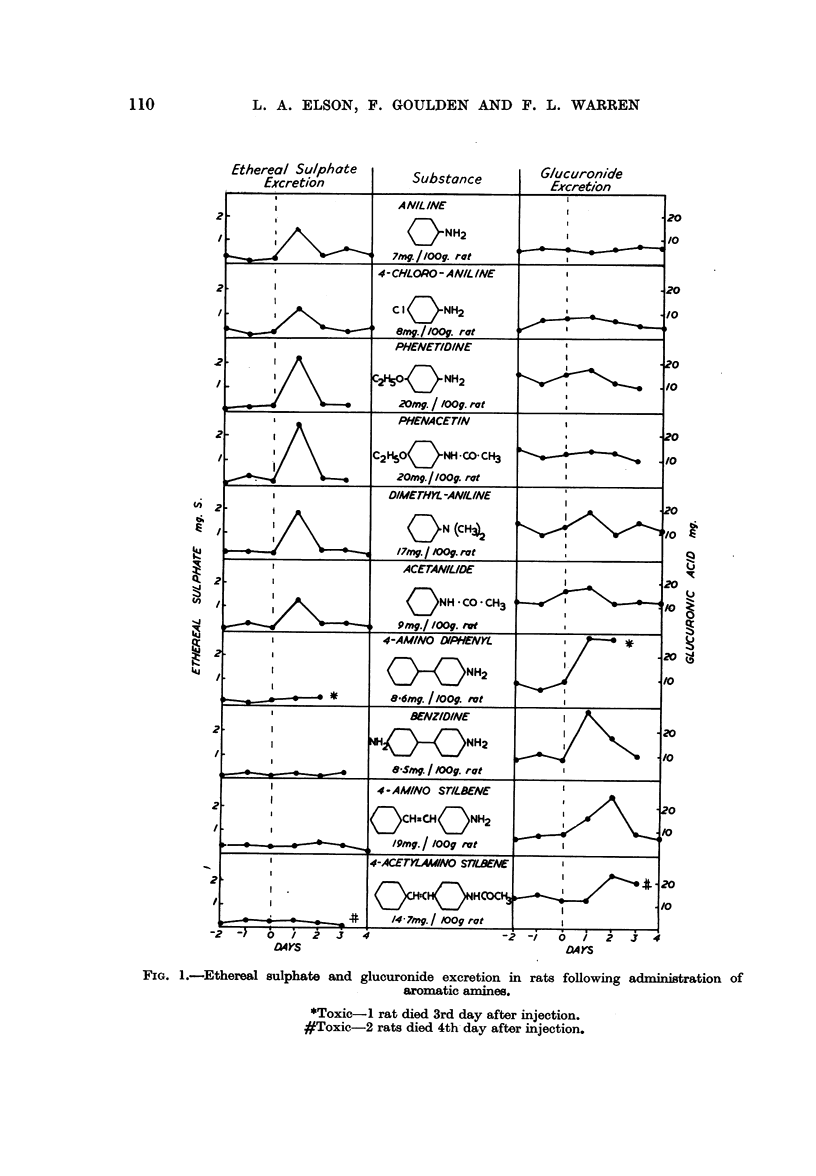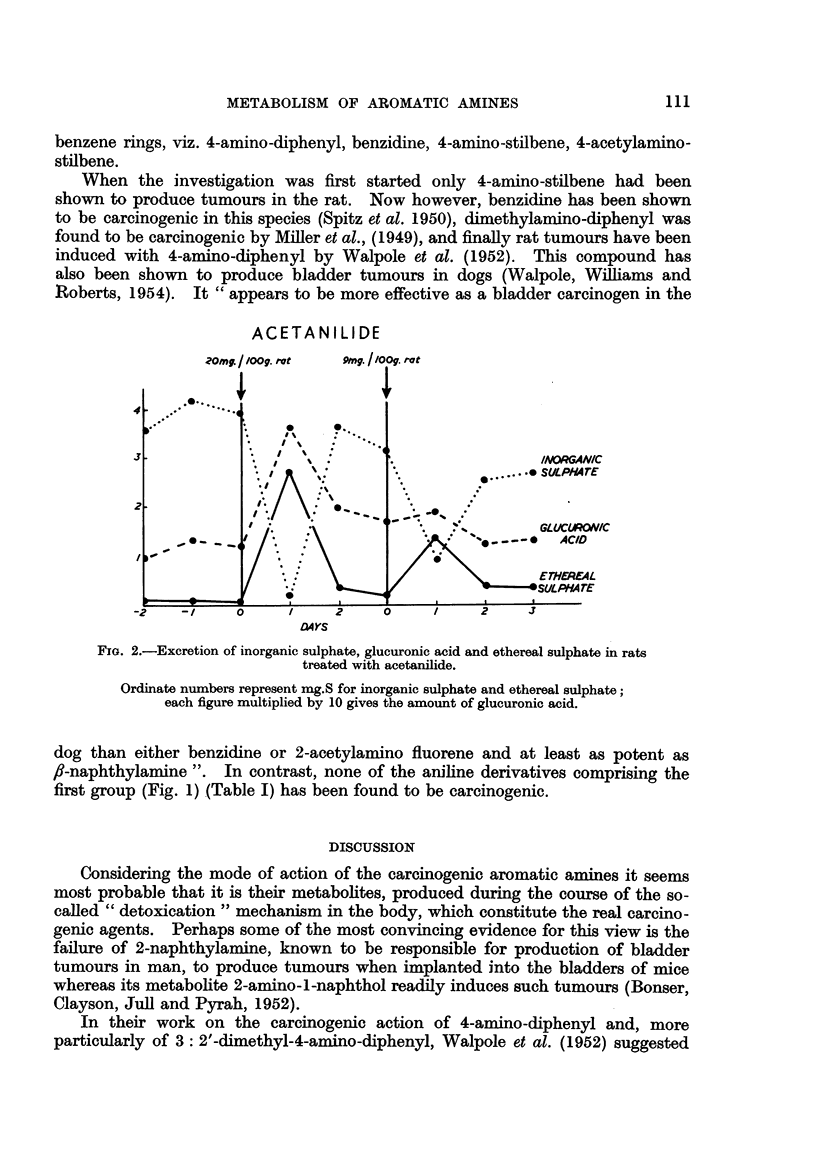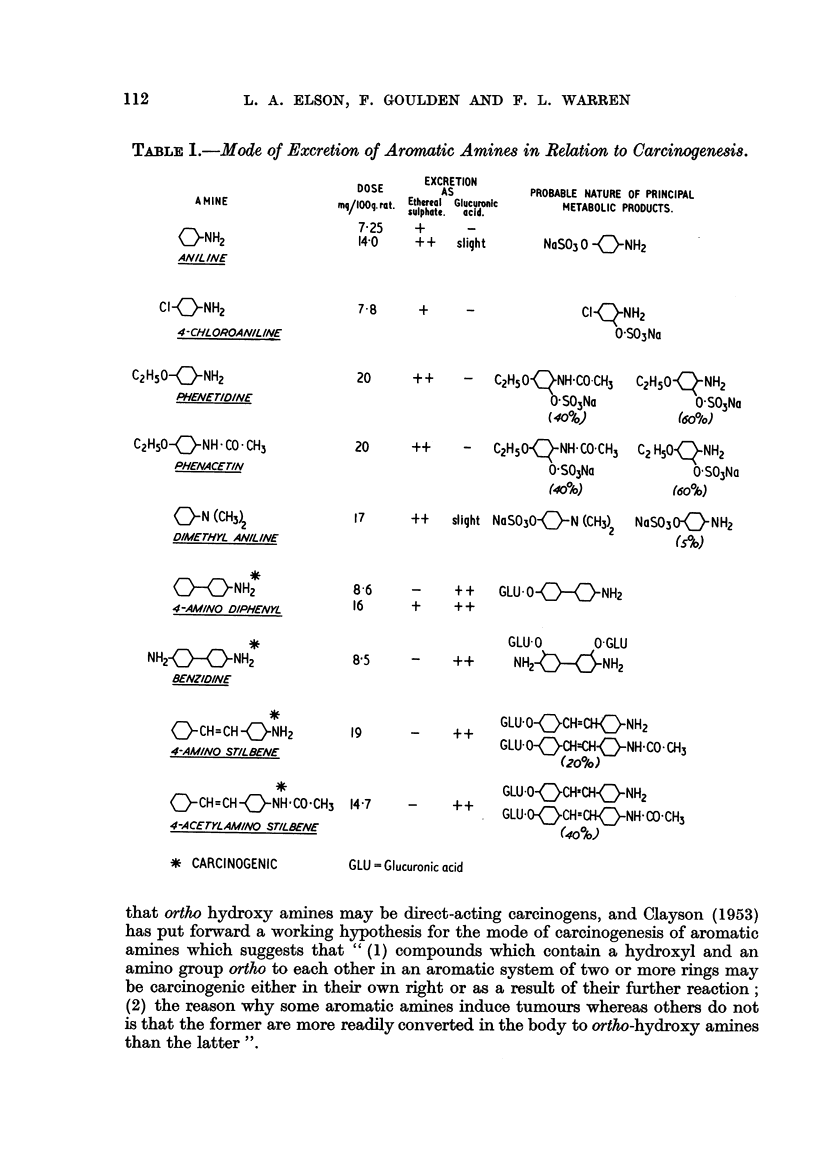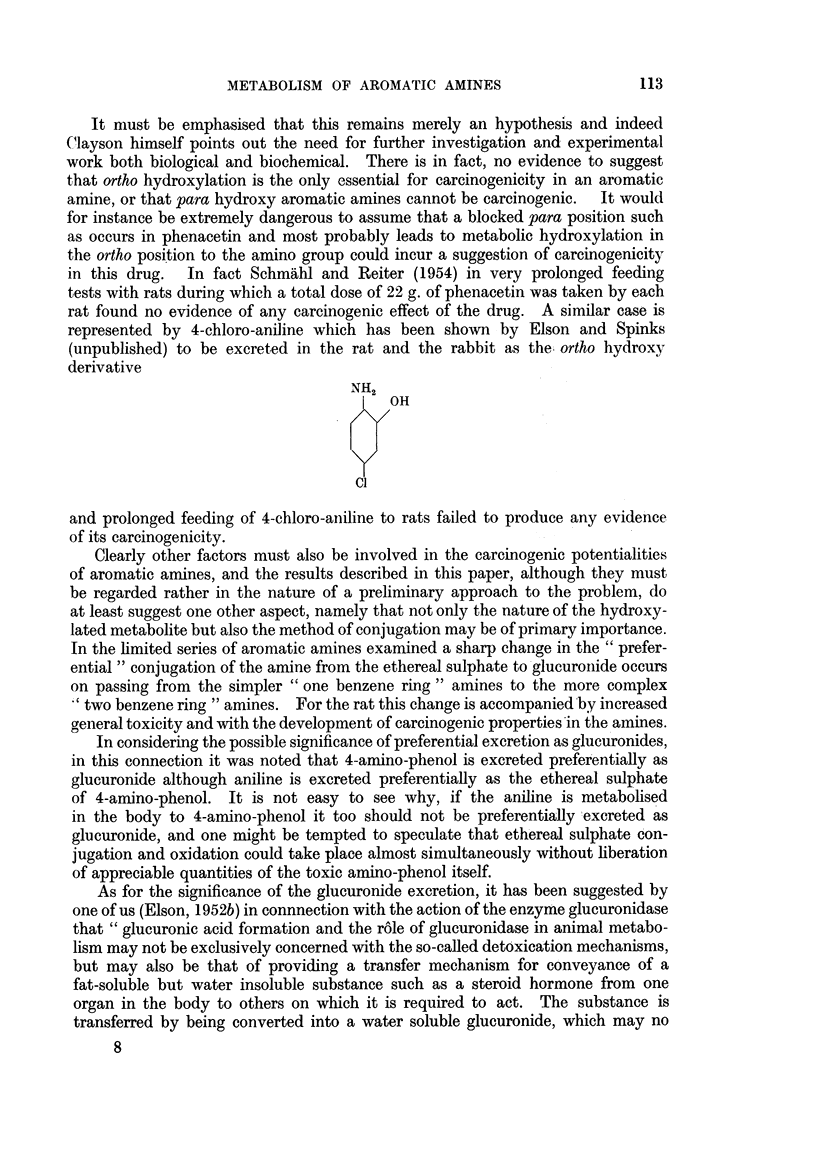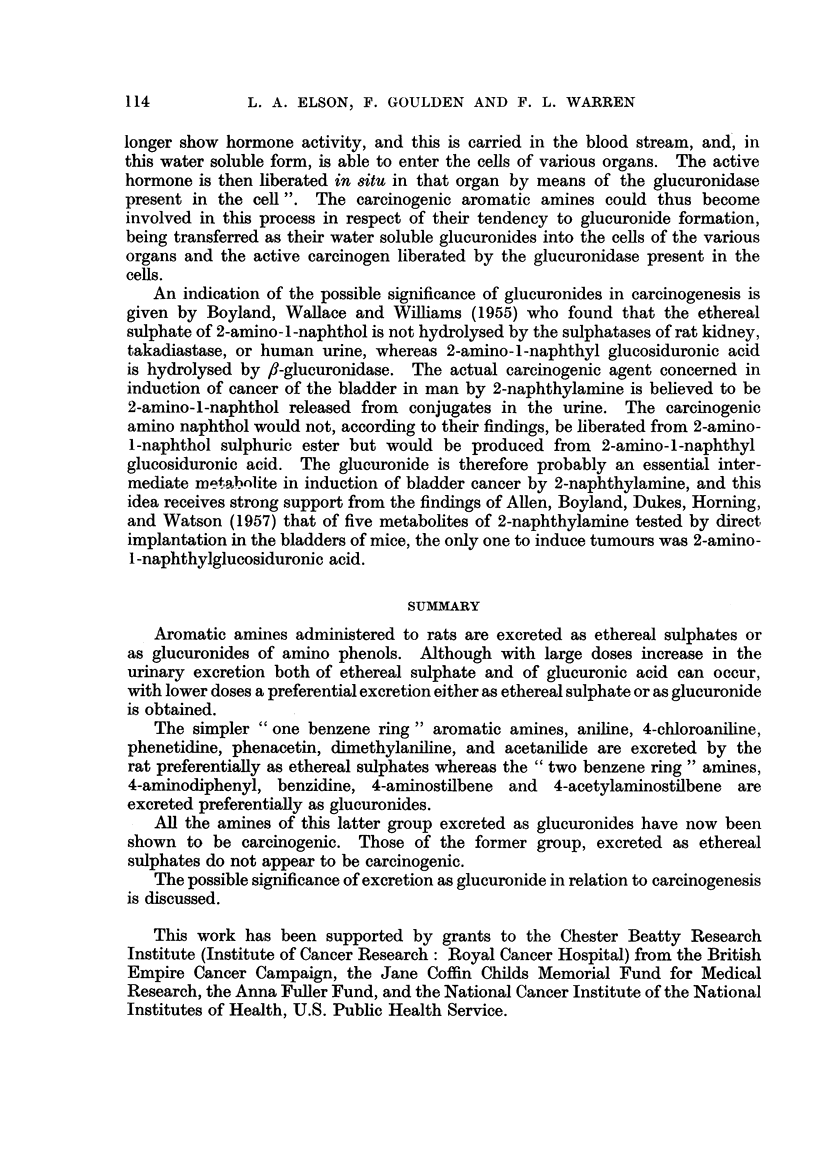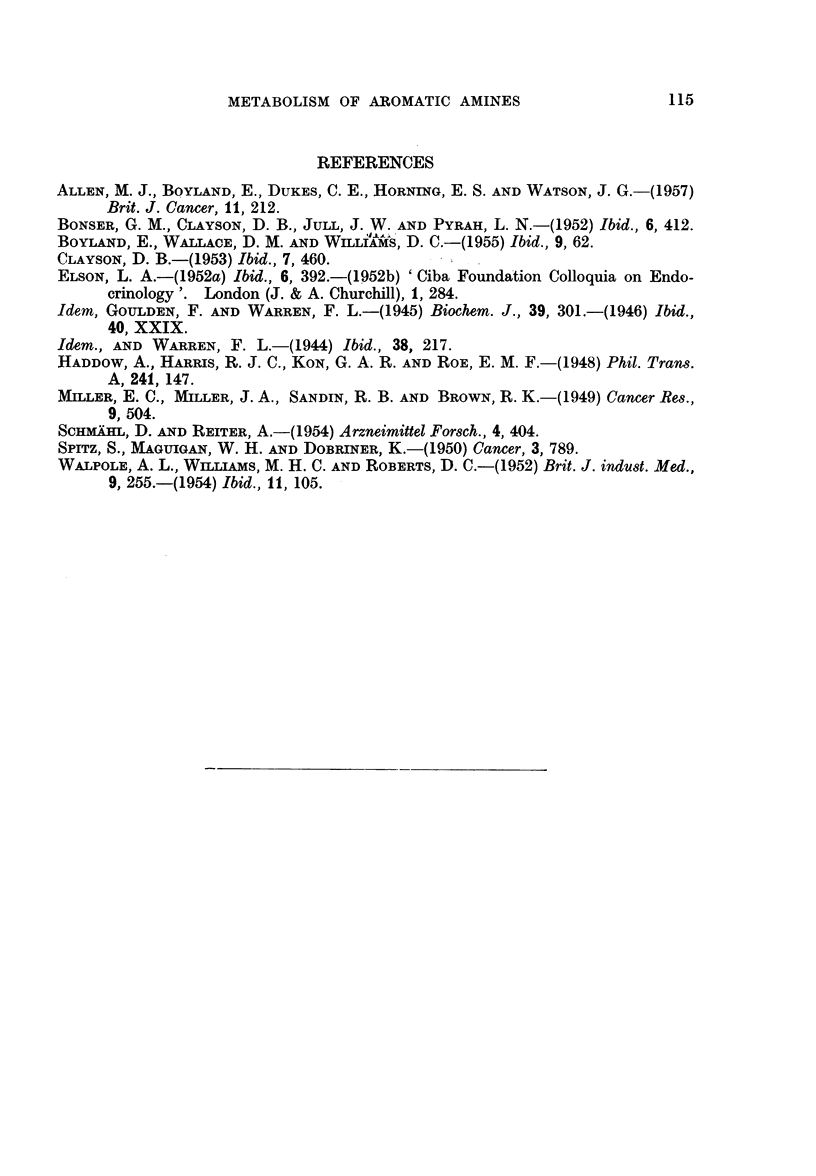# The Metabolism of Aromatic Amines in Relation to Carcinogenesis

**Published:** 1958-03

**Authors:** L. A. Elson, F. Goulden, F. L. Warren


### THE METABOLISM OF AROMATIC AMINES IN RELATION TO CARCINOGENESIS

L. A. ELSON, F. GOULDEN* AND F. L. WARREN†

*From the Chester Beatty Research Institute, The Royal Cancer Hospital, London, S.W.3*

Received for publication December 12, 1957.

SOME years ago, in connection with work on the metabolism of carcinogenic azo compounds, the method of excretion by the rat of a number of aromatic amines was studied. A preliminary account of some of this work was given at a meeting of the Biochemical Society (Elson, Goulden and Warren, 1946).

Many aromatic amines are excreted as ethereal sulphates of amino phenols, and one of the aims of this work was to investigate the effects of the amines on sulphur metabolism as had been done previously for a series of aromatic hydrocarbons (Elson, Goulden and Warren, 1945). The investigation was concerned therefore mainly with determining changes in the daily excretion of ethereal sulphate, inorganic sulphate, neutral sulphur and of glucuronic acid in the urine following administration of the aromatic amines.

It was hoped eventually to investigate more completely the metabolism of individual amines and to characterise definitely their metabolites, but this extended investigation has not been possible in most cases. The subsequent discovery of of the carcinogenic action in rats of a number of the amines, e.g. aminostilbenes (Haddow, Harris, Kon and Roe, 1948 ; Elson, 1952*a*), 4-dimethylamino-diphenyl (Miller, Miller, Sandin and Brown, 1949), benzidine (Spitz, Maguigan and Dobriner, 1950), and, in particular, 4-amino-diphenyl and 3 : 2′-dimethyl-4-aminodiphenyl by Walpole, Williams and Roberts (1952) and their suggestion that *ortho* hydroxy-amines may be direct-acting carcinogens, has suggested the possibility of an interesting relationship between our findings for the metabolism of these aromatic amines and their carcinogenic action.

The purpose of the present paper therefore is to describe in more detail the results of our metabolism studies and to discuss their possible significance in relation to carcinogenesis.

### EXPERIMENTAL

### *Animals*

The experiments were carried out on male rats maintained on a high protein diet (diet A, Elson 1952*a*). Groups of six rats were kept in one metabolism cage and supplied with diet and water *ad libitum.* The daily excretion of glucuronic acid, inorganic sulphate, ethereal sulphate, and in most cases neutral sulphur was determined as described by Elson, Goulden and Warren (1945). This was done for several days before administration of the amines dissolved in arachis

* Present address : Ashford Hospital, Middlesex.
† Present address : London Hospital Medical College, E.1.

oil, by intraperitoneal injection and the estimations were continued for several days subsequently. In some cases, 1 ml. arachis oil was injected prior to the control period of estimations preceding administration of the amine, but as this was found to give no appreciable changes in amounts of the excretion components estimated it was discontinued.

*Detection of free and conjugated amines*

In many cases the excretion of the amine was followed by a colorimetric method for detection of free aromatic amino groups by diazotisation and coupling with N-sulphato-ethyl-m-toluidine, as described by Elson and Warren (1944). By the use of the " Spekker " photoelectric absorptiometer for measuring the colour obtained with the untreated urine and the colour obtained after acid hydrolysis of the urine, it was often possible to obtain an idea of the amount of free and conjugated amine present. (An increase in the colour test after hydrolysis probably indicates that some of the original amine has been acetylated and excreted as the acetyl amino derivative, as is known to occur with drugs such as sulphonamides, etc.)

### RESULTS

The output of ethereal sulphates and glucuronides in the urine following injection of aromatic amines is shown in Fig. 1. With several of the amines the effect of more than one dose was investigated, and with some of them a large dose resulted in excretion of both ethereal sulphates and glucuronides. When the dose was reduced however, it was generally found that an increase of ethereal sulphate alone, or of glucuronide alone occurred, so that with moderate doses the amine showed a preferential excretion either as ethereal sulphate or as glucuronide. Fig. 1 shows the results obtained with these " preferential " doses.

A typical case where increased dosage results in excretion of glucuronide in addition to the " preferential " ethereal sulphate is shown in Fig. 2 which gives the results of acetanilide at doses of 20mg./100g. rat. and 9mg./100g. rat. The inorganic sulphate excretion is also shown and clearly demonstrates the fall in inorganic sulphate corresponding to the rise of ethereal sulphate.

Table I shows the mode of excretion of the amines in relation to their carcinogenic properties. The probable nature of the principal metabolic products is also suggested. In a number of cases the unconjugated metabolic products have been isolated and characterised by various workers. The other suggestions are based on general inferences considered in conjunction with the present results. The percentage figures under some of the formulae refer to the amine estimated colorimetrically by diazotisation and coupling with N-sulphato-ethyl-m-toluidine. Increase in the intensity of this colour test after hydrolysis probably indicates acetylation, and from the values before and after hydrolysis an estimate of the relative percentage of free and acetylated amine present in the original urine has been obtained.

From Fig. 1 it is seen that the amines fall clearly into two groups. The first group consists of compounds containing a single benzene ring, viz. aniline, 4-chloroaniline, phenetidine, dimethylaniline, and acetanilide, which are excreted preferentially as ethereal sulphates. The second group consists of amines excreted preferentially as glucuronides, and these are the ones containing two

FIG. 1.—Ethereal sulphate and glucuronide excretion in rats following administration of aromatic amines.

*Toxic—1 rat died 3rd day after injection.
#Toxic—2 rats died 4th day after injection.

benzene rings, viz. 4-amino-diphenyl, benzidine, 4-amino-stilbene, 4-acetylamino-stilbene.

When the investigation was first started only 4-amino-stilbene had been shown to produce tumours in the rat. Now however, benzidine has been shown to be carcinogenic in this species (Spitz *et al.* 1950), dimethylamino-diphenyl was found to be carcinogenic by Miller *et al.*, (1949), and finally rat tumours have been induced with 4-amino-diphenyl by Walpole *et al.* (1952). This compound has also been shown to produce bladder tumours in dogs (Walpole, Williams and Roberts, 1954). It " appears to be more effective as a bladder carcinogen in the

FIG. 2.—Excretion of inorganic sulphate, glucuronic acid and ethereal sulphate in rats treated with acetanilide.

Ordinate numbers represent mg.S for inorganic sulphate and ethereal sulphate; each figure multiplied by 10 gives the amount of glucuronic acid.

dog than either benzidine or 2-acetylamino fluorene and at least as potent as $\beta$-naphthylamine ". In contrast, none of the aniline derivatives comprising the first group (Fig. 1) (Table I) has been found to be carcinogenic.

### DISCUSSION

Considering the mode of action of the carcinogenic aromatic amines it seems most probable that it is their metabolites, produced during the course of the so-called " detoxication " mechanism in the body, which constitute the real carcinogenic agents. Perhaps some of the most convincing evidence for this view is the failure of 2-naphthylamine, known to be responsible for production of bladder tumours in man, to produce tumours when implanted into the bladders of mice whereas its metabolite 2-amino-1-naphthol readily induces such tumours (Bonser, Clayson, Jull and Pyrah, 1952).

In their work on the carcinogenic action of 4-amino-diphenyl and, more particularly of 3 : 2'-dimethyl-4-amino-diphenyl, Walpole *et al.* (1952) suggested

TABLE I.—*Mode of Excretion of Aromatic Amines in Relation to Carcinogenesis.*

| AMINE | DOSE mg/100g. rat. | EXCRETION AS Ethereal sulphate. | EXCRETION AS Glucuronic acid. | PROBABLE NATURE OF PRINCIPAL METABOLIC PRODUCTS. | |
|---|---|---|---|---|---|
|  ANILINE | 7·25<br>14·0 | +<br>++ | –<br>slight |  | |
|  4-CHLOROANILINE | 7·8 | + | – |  | |
|  PHENETIDINE | 20 | ++ | – |  (40%) |  (60%) |
|  PHENACETIN | 20 | ++ | – |  (40%) |  (60%) |
|  DIMETHYL ANILINE | 17 | ++ | slight |  |  (5%) |
|  4-AMINO DIPHENYL | 8·6<br>16 | –<br>+ | ++<br>++ |  | |
|  BENZIDINE | 8·5 | – | ++ |  | |
|  4-AMINO STILBENE | 19 | – | ++ |  (20%) | |
|  4-ACETYLAMINO STILBENE | 14·7 | – | ++ |  (40%) | |

* CARCINOGENIC GLU = Glucuronic acid that *ortho* hydroxy amines may be direct-acting carcinogens, and Clayson (1953) has put forward a working hypothesis for the mode of carcinogenesis of aromatic amines which suggests that " (1) compounds which contain a hydroxyl and an amino group *ortho* to each other in an aromatic system of two or more rings may be carcinogenic either in their own right or as a result of their further reaction ; (2) the reason why some aromatic amines induce tumours whereas others do not is that the former are more readily converted in the body to *ortho*-hydroxy amines than the latter ".

It must be emphasised that this remains merely an hypothesis and indeed Clayson himself points out the need for further investigation and experimental work both biological and biochemical. There is in fact, no evidence to suggest that *ortho* hydroxylation is the only essential for carcinogenicity in an aromatic amine, or that *para* hydroxy aromatic amines cannot be carcinogenic. It would for instance be extremely dangerous to assume that a blocked *para* position such as occurs in phenacetin and most probably leads to metabolic hydroxylation in the *ortho* position to the amino group could incur a suggestion of carcinogenicity in this drug. In fact Schmähl and Reiter (1954) in very prolonged feeding tests with rats during which a total dose of 22 g. of phenacetin was taken by each rat found no evidence of any carcinogenic effect of the drug. A similar case is represented by 4-chloro-aniline which has been shown by Elson and Spinks (unpublished) to be excreted in the rat and the rabbit as the *ortho* hydroxy derivative and prolonged feeding of 4-chloro-aniline to rats failed to produce any evidence of its carcinogenicity.

Clearly other factors must also be involved in the carcinogenic potentialities of aromatic amines, and the results described in this paper, although they must be regarded rather in the nature of a preliminary approach to the problem, do at least suggest one other aspect, namely that not only the nature of the hydroxylated metabolite but also the method of conjugation may be of primary importance. In the limited series of aromatic amines examined a sharp change in the " preferential " conjugation of the amine from the ethereal sulphate to glucuronide occurs on passing from the simpler " one benzene ring " amines to the more complex " two benzene ring " amines. For the rat this change is accompanied by increased general toxicity and with the development of carcinogenic properties in the amines.

In considering the possible significance of preferential excretion as glucuronides, in this connection it was noted that 4-amino-phenol is excreted preferentially as glucuronide although aniline is excreted preferentially as the ethereal sulphate of 4-amino-phenol. It is not easy to see why, if the aniline is metabolised in the body to 4-amino-phenol it too should not be preferentially excreted as glucuronide, and one might be tempted to speculate that ethereal sulphate conjugation and oxidation could take place almost simultaneously without liberation of appreciable quantities of the toxic amino-phenol itself.

As for the significance of the glucuronide excretion, it has been suggested by one of us (Elson, 1952*b*) in connnection with the action of the enzyme glucuronidase that " glucuronic acid formation and the rôle of glucuronidase in animal metabolism may not be exclusively concerned with the so-called detoxication mechanisms, but may also be that of providing a transfer mechanism for conveyance of a fat-soluble but water insoluble substance such as a steroid hormone from one organ in the body to others on which it is required to act. The substance is transferred by being converted into a water soluble glucuronide, which may no longer show hormone activity, and this is carried in the blood stream, and, in this water soluble form, is able to enter the cells of various organs. The active hormone is then liberated *in situ* in that organ by means of the glucuronidase present in the cell ". The carcinogenic aromatic amines could thus become involved in this process in respect of their tendency to glucuronide formation, being transferred as their water soluble glucuronides into the cells of the various organs and the active carcinogen liberated by the glucuronidase present in the cells.

An indication of the possible significance of glucuronides in carcinogenesis is given by Boyland, Wallace and Williams (1955) who found that the ethereal sulphate of 2-amino-1-naphthol is not hydrolysed by the sulphatases of rat kidney, takadiastase, or human urine, whereas 2-amino-1-naphthyl glucosiduronic acid is hydrolysed by $\beta$-glucuronidase. The actual carcinogenic agent concerned in induction of cancer of the bladder in man by 2-naphthylamine is believed to be 2-amino-1-naphthol released from conjugates in the urine. The carcinogenic amino naphthol would not, according to their findings, be liberated from 2-amino-1-naphthol sulphuric ester but would be produced from 2-amino-1-naphthyl glucosiduronic acid. The glucuronide is therefore probably an essential intermediate metabolite in induction of bladder cancer by 2-naphthylamine, and this idea receives strong support from the findings of Allen, Boyland, Dukes, Horning, and Watson (1957) that of five metabolites of 2-naphthylamine tested by direct implantation in the bladders of mice, the only one to induce tumours was 2-amino-1-naphthylglucosiduronic acid.

### SUMMARY

Aromatic amines administered to rats are excreted as ethereal sulphates or as glucuronides of amino phenols. Although with large doses increase in the urinary excretion both of ethereal sulphate and of glucuronic acid can occur, with lower doses a preferential excretion either as ethereal sulphate or as glucuronide is obtained.

The simpler " one benzene ring " aromatic amines, aniline, 4-chloroaniline, phenetidine, phenacetin, dimethylaniline, and acetanilide are excreted by the rat preferentially as ethereal sulphates whereas the " two benzene ring " amines, 4-aminodiphenyl, benzidine, 4-aminostilbene and 4-acetylaminostilbene are excreted preferentially as glucuronides.

All the amines of this latter group excreted as glucuronides have now been shown to be carcinogenic. Those of the former group, excreted as ethereal sulphates do not appear to be carcinogenic.

The possible significance of excretion as glucuronide in relation to carcinogenesis is discussed.

This work has been supported by grants to the Chester Beatty Research Institute (Institute of Cancer Research : Royal Cancer Hospital) from the British Empire Cancer Campaign, the Jane Coffin Childs Memorial Fund for Medical Research, the Anna Fuller Fund, and the National Cancer Institute of the National Institutes of Health, U.S. Public Health Service.

### REFERENCES

ALLEN, M. J., BOYLAND, E., DUKES, C. E., HORNING, E. S. AND WATSON, J. G.—(1957) *Brit. J. Cancer*, **11**, 212.

BONSER, G. M., CLAYSON, D. B., JULL, J. W. AND PYRAH, L. N.—(1952) *Ibid.*, **6**, 412.

BOYLAND, E., WALLACE, D. M. AND WILLIAMS, D. C.—(1955) *Ibid.*, **9**, 62.

CLAYSON, D. B.—(1953) *Ibid.*, **7**, 460.

ELSON, L. A.—(1952*a*) *Ibid.*, **6**, 392.—(1952b) ' Ciba Foundation Colloquia on Endocrinology '. London (J. & A. Churchill), **1**, 284.

*Idem*, GOULDEN, F. AND WARREN, F. L.—(1945) *Biochem. J.*, **39**, 301.—(1946) *Ibid.*, **40**, XXIX.

*Idem.*, AND WARREN, F. L.—(1944) *Ibid.*, **38**, 217.

HADDOW, A., HARRIS, R. J. C., KON, G. A. R. AND ROE, E. M. F.—(1948) *Phil. Trans.* A, **241**, 147.

MILLER, E. C., MILLER, J. A., SANDIN, R. B. AND BROWN, R. K.—(1949) *Cancer Res.*, **9**, 504.

SCHMÄHL, D. AND REITER, A.—(1954) *Arzneimittel Forsch.*, **4**, 404.

SPITZ, S., MAGUIGAN, W. H. AND DOBRINER, K.—(1950) *Cancer*, **3**, 789.

WALPOLE, A. L., WILLIAMS, M. H. C. AND ROBERTS, D. C.—(1952) *Brit. J. indust. Med.*, **9**, 255.—(1954) *Ibid.*, **11**, 105.